# Risk-Adaptive Volumetric Modulated Arc Therapy Using Biological Objective Functions for Subvolume Boosting in Radiotherapy

**DOI:** 10.1155/2012/348471

**Published:** 2012-06-27

**Authors:** Nicholas Hardcastle, Wolfgang A. Tome

**Affiliations:** ^1^Departments of Human Oncology and Medical Physics, University of Wisconsin-Madison, Madison, WI 53792, USA; ^2^Department of Physical Sciences, Peter MacCallum Cancer Centre, East Melbourne, VIC 3002, Australia; ^3^Centre for Medical Radiation Physics, University of Wollongong, Wollongong, NSW 2522, Australia; ^4^Oncophysics Institute, Albert Einstein College of Medicine of Yeshiva University, Jack and Pearl Resnick Campus, 1300 Morris Park Avenue, Bronx, NY 10461, USA

## Abstract

*Objectives*. Simultaneous integrated boost (SIB) for prostate cancer allows increases in tumor control probability while respecting normal tissue dose constraints. Biological optimization functions that optimize based on treatment outcome can be used to create SIB prostate plans. This study investigates the feasibility of biologically optimized volumetric modulated arc therapy (VMAT) for SIB prostate radiotherapy. *Methods*. Five prostate cancer patients with diffusion-weighted MR images were selected for analysis. A two-step VMAT optimization was performed, which consisted of an initial biological optimization of a static gantry angle delivery followed by conversion of the static delivery to a single arc VMAT plan. A dosimetric analysis was performed on the resulting plans. *Results*. The VMAT plans resulted in a ΔEUD between the prostate and the boost volume of between 15.1 Gy and 20.3 Gy. Rectal volumes receiving 75.6 Gy ranged from 4.5 to 9.9%. Expected rectal normal tissue complication probabilities were between 8.6% and 21.4%. Maximum bladder doses ranged from 73.6 Gy to 75.8 Gy. Estimated treatment time was 120 s or less. *Conclusions*. The presented biological optimization method resulted in deliverable VMAT plans that achieved sufficient modulation for SIB without violating rectal and bladder dose constraints. *Advances in knowledge*. This study presents a method for creating simultaneous integrated boost VMAT treatments using biological outcome objective functions.

## 1. Introduction

The benefit of a simultaneous integrated boost (SIB) for prostate cancer is that it allows one to increase the expected tumor control probability (TCP) while at the same time respecting normal tissue dose constraints, compared to the whole prostate dose escalation for plans delivering the same equivalent uniform dose (EUD) to the entire prostate [1, 2]. When delivering an SIB for prostate cancer, biological objective functions for optimization of dose distributions, as opposed to physical dose objective functions can be utilized. That is, direct optimization of biological outcomes can be performed. Using this technique, it has been shown that, for prostate cancer, SIB can be delivered to a subvolume within the prostate while adhering to normal tissue constraints. [1, 3] SIB techniques allow one to take advantage of advanced imaging techniques to selectively boost the dose to a subvolume of the treatment target that may not be controlled with standard doses and hence require a dose higher than the minimal peripheral prescription dose to be delivered to them to achieve adequate local control.

SIB delivery requires increased modulation of the dose so as to achieve the desired boosting level while maintaining a sufficient dose gradient outside of the target to minimize the dose to surrounding healthy tissues. Kim and Tomé have shown that static gantry angle IMRT can achieve the required modulation [1, 3–5]. In recent years, volumetric modulated arc therapy (VMAT) has become an attractive method to efficiently deliver modulated dose distributions. VMAT has been shown to result in equivalent dose distributions in prostate cancer in shorter delivery times when compared with static gantry angle intensity modulated radiation therapy (IMRT) [6–8]. Biological optimization of VMAT plans for prostate cancer has also been investigated [6, 9]. The vast majority of these studies have concentrated on prostate only or prostate plus seminal vesicles as the target volume. It can be argued that achievement of sufficient modulation in these cases is more accessible than for more complex treatment geometries. Jolly et al. [10] have provided a robust planning strategy for an SIB technique with VMAT, treating to three dose levels including the prostate and seminal vesicles according to the Conventional or Hypofractionated High-Dose Intensity-Modulated Radiotherapy for Prostate Cancer (CHHiP) protocol [11]. The volume receiving the highest dose under the CHHiP protocol, however, is the full prostate plus a 5 mm margin, so the complexity of these targets is limited. The present study extends the investigation of VMAT for SIB to a more complex geometry, by using a risk-adaptive VMAT optimization method, where the boost volume is a subvolume of the prostate.

## 2. Methods and Materials

Five patients were selected for analysis. These included two patients using rectal balloons and three patients without rectal balloons. All the patients received a planning kVCT scan as well as a T2 contrast-enhanced magnetic resonance (MR) image. The prostate was contoured as the CTV, which was expanded by 7 mm to obtain the PTV. A biopsy-confirmed subvolume of enhanced contrast uptake on the diffusion weighted imaging (DWI) MR was outlined as the high-risk CTV. The high-risk CTV plus 8 mm was subtracted from the PTV to obtain the residual PTV, PTV-R. A 5 mm expansion was applied to the high-risk CTV to obtain the high-risk PTV, PTV-H. This process resulted in no overlap between PTV-R and PTV-H. The rectal wall was outlined on the patients with rectal balloon, and the full rectum was outlined on the patients with no rectal balloon. The bladder and femoral heads were also outlined. An avoidance structure was outlined, which consisted of a 5 cm wide ring around the PTV plus 2 cm.

All planning was performed with the Pinnacle radiotherapy planning system using the SmartArc VMAT optimization algorithm (v9.100, Philips Radiation Oncology Systems, Fitchburg, WI, USA). A set of research optimization objectives, built into the Pinnacle RTPS as a plugin, that optimize directly on TCP and NTCP were used. The plugin and objective function have been described in detail elsewhere [3]. Briefly, the objective function maximizes the TCP for target volumes while minimizing the NTCP for organs at risk (OARs). The objective function takes the form of

(1)UTCP=TCP(1−NTCP).

The TCP and NTCP calculations utilize phenomenological logistic dose response functions that are evaluated on a voxel-by-voxel basis. That is, the TCP and NTCP are calculated for each voxel, where each voxel has its own value of *D*_50_ and *γ*_50_ (for TCP) and *D*_50_ and *m* (for NTCP) based on the physiological makeup of the tumor. *D*_50_ is the dose that yields a tumor control probability or normal tissue complication probability of 50% for TCP and NTCP respectively, and *γ*_50_ and *m* are parameters describing the slope of the dose response curve at the 50% probability point for TCP and NTCP, respectively. TCP and NTCP are calculated on a voxel-by-voxel basis so as to be able to account for subtumor variations in the dose response curves for tumor control and normal tissue complication. In this study, the tumor is divided into two subvolumes requiring separate parameters to describe their dose response curve, and normal structures are approximated as one subvolume. The reader is referred to Kim and Tomé for details and derivation of the TCP and NTCP functions. [3]

A two-step process was used for optimization. In the first step, a set of 15 beams were distributed every 24° around the patient. These were selected to mimic the positions of the initial fluence maps used in the SmartArc optimization process. Intensity modulation of the ideal fluence was then performed using the biological optimization function described above. The optimization objectives for each organ and target are given in Table 1. TCP objectives were used on the PTV-H and the PTV-R. In addition to these TCP objectives on the target, an NTCP objective was used on the original PTV to limit the PTV dose to less than 100 Gy. Two NTCP objectives were used on the rectum. The first used the values of *D*_50_ and *m* representing grade 2 or higher rectal bleeding from the QUANTEC study [12].Since the QUANTEC values are related to grade 2 or higher rectal bleeding these tended to penalize only the high dose region of the rectum. Therefore, a second NTCP objective function was used to reduce the mid-low doses, the values of which were taken from the NTCP parameter fitting by Tucker et al. [13]. We note that although these values were derived also for grade 2 or higher rectal bleeding, the parameter values penalize mid-low doses thus were used as a surrogate for rectal toxicities related to mid-low doses.

The optimization was run until the objective value was reduced below a given value, typically 5–10 iterations. The second step of the optimization involved creating a single arc VMAT plan using the SmartArc optimization tool and an Elekta Infinity linear accelerator with the X MLC for delivery (Elekta North America, Atlanta, GA, USA). A 360° arc was selected, with a collimator angle of 30° and dose rate set to be discretely variable from 0 to 300 MU/min. The final gantry spacing was 4°, and the maximum delivery time was 120 seconds. Physical dose optimization objectives were created based on the ideal dose distribution derived from the biological optimization. Two steps of 40 iterations were performed. Dose calculation based on a 2 × 2 × 2 mm^3^ dose grid was then performed using the adaptive convolution dose algorithm. The rationale for the two-step optimization process was to allow the optimizer to create an “ideal” set of fluence maps, which would adhere to the optimization objectives as close as possible, before using this “ideal” dose distribution as the basis for the deliverable SmartArc optimization.That is, the initial biological optimization allows one to obtain the highest boost dose possible to the subtumor volume without violating the normal tissue constraints on the normal tissue volumes. 

 The plans were the transferred to Computational Environment for Radiotherapy Research (CERR, University of Washington in St. Louis) for analysis [14]. For all plans, the following metrics were calculated: EUD of the PTV-H and PTV-R and the difference between these values ΔEUD, percentage volume of the rectum receiving 25 Gy, 50 Gy, and 70 Gy, the rectal NTCP (calculated using the QUANTEC values for ≥ grade 2 rectal bleeding of  *D*_50_ = 76.9 Gy, *m* = 0.13, and *n* = 0.09), the bladder volume receiving 55 Gy, and the maximum bladder dose. The maximum bladder dose was taken as the dose 1 cc of the bladder receiving the highest dose.EUD was calculated using Equation 8 in [15] with an SF_2_ of 0.48. 

In addition to the plan quality analysis, one treatment plan was selected for delivery quality assurance on an Elekta Infinity linear accelerator. The treatment plan for patient 3 was calculated on a kVCT of the Delta4 phantom (Scandidos, Uppsala, Sweden). The plan was delivered and the dose was measured in the phantom. Comparison of the delivered dose with the planned dose was performed using the 2D gamma metric with 3%/3 mm criteria. 

## 3. Results 

**Figure 1:**
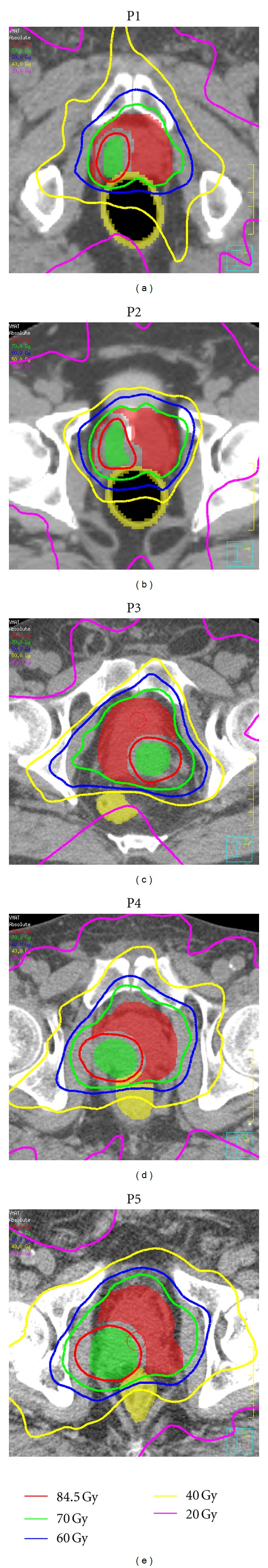
Isodose lines for the five patients. PTV-H is the green colorwash, PTV-R is the red colorwash and the rectum is the yellow colorwash. P1 and P2 each had a rectal balloon; P3, P4, and P5 had no balloon.

**Figure 2:**
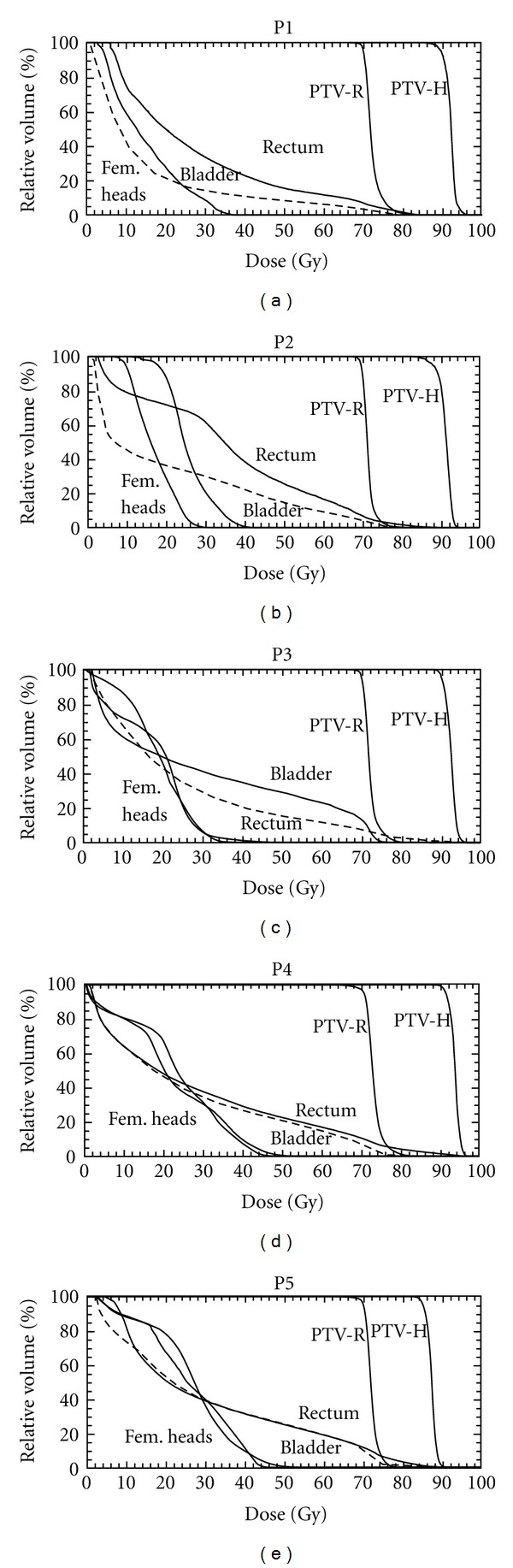
DVHs for the five patients. The bladder line is dashed.

The dose distributions and metrics were compared for the five patients.  Figure 1 shows the isodose lines for the five patients, showing that sufficient modulation was achieved with one arc to conform the boost dose to the PTV-H volume with limited coverage of the rectal volume with the boost dose.  Figure 2 shows the DVHs for the five patients for both the PTVs and the OARs. 

 The dosimetric metrics are presented in Table 2. The PTV-H targets received EUDs of between 88.34 Gy and 94.75 Gy; however, these had an upper dosimetric limit placed on them during optimization, without which dose volume histograms (DVHs) would exceed 100 Gy. Subtumor boosting allowed an increase in EUD to the high-risk PTV of between 15.22 Gy and 20.55 Gy over the dose the PTV-R received. 

 The rectal DVH values for all patients met the V50 Gy < 30% objective presented by Mohan et al. for the 2.5 Gy × 28 fractions regime [16]. Due to the increased doses per fraction used in this planning study, the maximum rectal dose objective of 74 Gy used by Mohan et al. was not considered [16]. Instead, the volumes receiving 75.6 Gy were analysed as per the 86.4 Gy treatment regime reported by Zelefsky et al. [17]. All patients easily met this treatment objective of V75.6 Gy < 30%, with the maximum being 6.3% (patient 5). The rectal NTCP values for ≥grade 2 rectal bleeding range from 8.2% to 20.0%. The bladder DVH values all met the V55 Gy < 30% specified by Mohan et al. [16]. There was no discernible dosimetric difference between the two patients with balloons and the three patients without balloons. 

**Figure 3:**
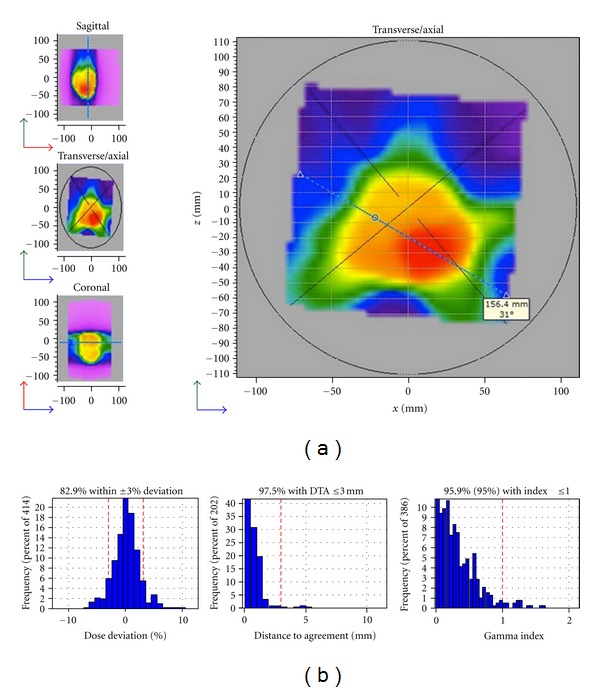
VMAT delivery QA for patient 3. (a) shows the axial distribution obtained by delivering a risk adaptive VMAT plan to the Delta4 phantom. (b) shows the respective distributions for the dose deviation, distance to agreement, and gamma index found for this plan.

 The measured dose in the Delta4 phantom was compared with the planned dose for the treatment plan selected for delivery. Using a gamma criteria of 3%/3 mm, 95.9% of all points passed (cf.  Figure 3(b)). 

## 4. Discussion 

The capability of VMAT for achieving sufficient modulation for prostate radiotherapy with simultaneous integrated boost has been investigated in this study. Despite the more complex target geometry, one treatment arc was sufficient to meet treatment objectives. This allows one to take advantage of advanced imaging techniques such as MRI and PET to identify subregions in the heterogeneous tumor environment and efficiently escalate the dose to selected volumes. It should be noted that this technique could potentially be applied to other treatment sites where simultaneous integrated boosting could be advantageous. 

Despite the large differences in the rectal anatomy between patients with a rectal balloon and patients without a rectal balloon, there was very little difference in the planned rectal dose between patients with and without a rectal balloon. However, due to the high doses delivered to the PTV-H volume, which is often in the posterior lobe of the prostate, adjacent to the rectum, any small changes in prostate or rectal position, size or shape could lead to large differences between the planned and delivered doses. As a result, use of a rectal balloon would be beneficial due to the improved setup reproducibility and consistency of the rectal size and shape provided by the use of a rectal balloon. 

Although contoured, the bladder and femoral head structures were not used for optimization due to their having very little impact on the optimization result. That is to say, optimization on these structures was not required to obtain the low bladder and femoral head doses observed here. This is likely a consequence of the combination of delivery technique, where the dose is delivered from all angles which spreads out the dose distribution, minimizing the dose delivered to each critical structure, and the optimization using the normal structure, which has the effect of steepening the dose gradient outside of the target volume. 

The prostate dose in the presented plans was limited by treating the PTV-H and PTV-R as target volumes but the whole PTV as a normal tissue structure. This is akin to the method for optimizing target dose based on the EUD function presented by Wu et al. [18], who treated the target volume as both a target and a normal tissue structure to improve the homogeneity of the dose distribution. The result of our target optimization method was that the dose to the PTV-H was boosted to an EUD of 88–95 Gy whilst still maintaining an EUD between 72.5 Gy and 74.2 Gy to the PTV-R. This limits the dose to the urethra and rectum to doses less than that allowed in the MSKCCC dose escalation trial. [17] Our current method differs from that presented by Kim and Tomé [3], who did not limit the dose on the prostate and as such observed a more heterogeneous target dose. The dose to the normal structures using the VMAT technique was less than that achieved by Kim and Tomé [3] who used a static gantry angle IMRT technique.The current method also used two NTCP objectives on the rectum (as opposed to one in Kim and Tomé [3]) to reduce the rectal volumes receiving both high and mid-low doses. This is also a contributing factor to the reduced rectal doses observed in the current study. 

## 5. Conclusions 

We have shown that biologically optimized VMAT plans can be derived from a risk-adaptive subtumor dose escalation strategy for prostate cancer. A single arc provides sufficient modulation to achieve dose escalation to a high-risk subvolume of the prostate of up to a ΔEUD of 20.3 Gy above the gEUD of the remainder of the prostate. This was achieved without violating rectal NTCP constraints, allowing for efficient delivery of risk-adaptive prostate radiotherapy. 

## Figures and Tables

**Table 1:** Biological objective functions and parameters.

ROI	Objective	Dose	*m*	*γ*
PTV	NTCP	95	0.11	—
PTV-H	TCP	84.5	—	8
PTV-R	TCP	64.5	—	8
Rectum	NTCP	76.9	0.13	—
Rectum	NTCP	55.9	0.15	—
Normal	NTCP	55	0.13	—

**Table 2:** Dosimetric metrics for the target and OAR structures.

Patient	PTV-H	PTV-R	ΔEUD	Rectum				Bladder	
	EUD (Gy)	EUD (Gy)		V50 Gy (%)	V70 Gy (%)	V75.6 Gy (%)	NTCP (%)	V55 Gy (%)	Max (Gy)
P1	92.8	72.9	19.9	17.2	8.7	5.2	11.1	7.4	78.7
P2	92.2	72.5	19.7	26.5	7.5	3.2	8.2	11.8	74.5
P3	93.3	72.9	20.3	15.3	7.5	3.8	9.9	26.0	74.9
P4	94.8	74.2	20.6	22.8	11.0	6.2	20.0	17.5	75.6
P5	88.3	73.1	15.2	25.4	12.3	6.3	13.0	23.0	74.1
